# Retest variability and patient reliability indices of quantitative fundus autofluorescence in age-related macular degeneration: a MACUSTAR study report

**DOI:** 10.1038/s41598-023-43417-y

**Published:** 2023-10-13

**Authors:** Leon von der Emde, Merten Mallwitz, Marc Vaisband, Jan Hasenauer, Marlene Saßmannshausen, Jan Henrik Terheyden, MACUSTAR Consortium, H. Agostini, L. Altay, R. Atia, F. Bandello, P. G. Basile, J. Batuca, C. Behning, M. Belmouhand, M. Berger, A. Binns, C. J. F. Boon, M. Böttger, C. Bouchet, J. E. Brazier, T. Butt, C. Carapezzi, J. Carlton, A. Carneiro, A. Charil, R. Coimbra, M. Cozzi, D. P. Crabb, J. Cunha-Vaz, C. Dahlke, L. de Sisternes, H. Dunbar, R. P. Finger, E. Fletcher, H. Floyd, C. Francisco, M. Gutfleisch, S. Hinz, R. Hogg, F. G. Holz, C. B. Hoyng, A. Kilani, J. Krätzschmar, L. Kühlewein, M. Larsen, S. Leal, Y. T. E. Lechanteur, U. F. O. Luhmann, A. Lüning, I. Marques, C. Martinho, G. Montesano, Z. Mulyukov, M. Paques, B. Parodi, M. Parravano, S. Penas, T. Peters, T. Peto, M. Pfau, S. Poor, S. Priglinger, D. Rowen, G. S. Rubin, J. Sahel, C. Sánchez, O. Sander, M. Saßmannshausen, M. Schmid, S. Schmitz-Valckenberg, J. Siedlecki, R. Silva, A. Skelly, E. Souied, G. Staurenghi, L. Stöhr, D. J. Taylor, J. H. Terheyden, S. Thiele, A. Tufail, M. Varano, L. Vieweg, L. Wintergerst, A. Wolf, N. Zakaria, Kenneth R. Sloan, Steffen Schmitz-Valckenberg, Robert P. Finger, Frank G. Holz, Thomas Ach

**Affiliations:** 1 Department of Ophthalmology, University Hospital Bonn, University of Bonn, Ernst-Abbe-Straße 2, 53127 Bonn, Germany; 2 Life & Medical Sciences Institute, University of Bonn, Bonn, Germany; 3 Department of Internal Medicine III with Haematology, Medical Oncology, Haemostaseology, Infectiology and Rheumatology, Oncologic Center, Paracelsus Medical University, Salzburg, Austria; 4 Helmholtz Center Munich-German Research Center for Environmental Health, Institute of Computational Biology, Neuherberg, Germany; 5 Universitaetsklinikum Freiburg (UKLFR), Department of Ophthalmology, University of Freiburg, Freiburg, Germany; 6 Department of Ophthalmology, University Hospital of Cologne, Cologne, Germany; 7 Quinze-Vingts National Ophthalmology Hospital, UPMC-Sorbonne Université, Paris, France; 8 Department of Ophthalmology, University Vita Salute-Scientific Institute of San Raffael, Milan, Italy; 9 AIBILI Association for Innovation and Biomedical Research on Light and Image (AIBILI), Coimbra, Portugal; 10 GRADE Reading Center, University of Bonn, Bonn, Germany; 11 Department of Ophthalmology, Rigshospitalet-Glostrup, Copenhagen University, Glostrup, Denmark; 12 Institute for Medical Biometry, Informatics and Epidemiology, University of Bonn, Bonn, Germany; 13 City University London, London, UK; 14 Department of Ophthalmology, Leiden University Medical Center, Leiden, The Netherlands; 15 BAYER AG, Leverkusen, Germany; 16 Novartis Pharma AG, Basel, Switzerland; 17 University of Sheffield, Sheffield, UK; 18 University College London (UCL), London, UK; 19 Fondation Voir et Etendre, Paris, France; 20 Department of Ophthalmology, Centro Hospitalar de Sao Joao EPE (Hospital Sao Joao), Porto Medical School, Porto, Portugal; 21 Department of Ophthalmology Luigi Sacco Hospital, University of Milan, Milan, Italy; 22 Carl Zeiss Meditec, AG, Jena, Germany; 23 Department of Ophthalmology, University of Bonn, Venusberg-Campus 1, 53127 Bonn, Germany; 24 Clinical Trial Unit, Department of Ophthalmology, Gloucestershire Hospitals NHS Foundation Trust, Cheltenham, UK; 25 Department of Ophthalmology, St. Franziskus Hospital, Münster, Germany; 26 STZ Biomed and STZ Eyetrial at the Center of Ophthalmology, University Hospital Tuebingen, Tübingen, Germany; 27 Stichting Katholieke Universiteit/Radboud University Medical Center (SRUMC), Radbound University, Nijmegen Medical Center, Nijmegen, The Netherlands; 28 F. Hoffmann-La Roche Ltd, Basel, Switzerland; 29 G. B. Bietti Eye Foundation-IRCCS, Rome, Italy; 30 Ophthalmology and Vision Sciences, The Queen’s University and Royal Group of Hospitals Trust, Belfast, Northern Ireland UK; 31 Ophthalmic Genetics and Visual Function Branch, National Eye Institute, Bethesda, MD USA; 32 Ludwig-Maximilians-Universitaet Muenchen (LMU), University Eye Hospital, Munich, Germany; 33 Centre Hospitalier National d’Opthalmologie des Quinze-Vingts, Paris, France; 34 Department of Ophthalmology and Visual Sciences, John A. Moran Eye Center, University of Utah, Salt Lake City, UT USA; 35 Centre Hospitalier Intercommunal de Creteil (HIC), University Eye Clinic, Centre Hospitalier Creteil, Paris, France; 36 European Clinical Research Infrastructure Network (ECRIN), Paris, France; 37 Moorfields Eye Hospital NHS Foundation Trust (MBRC), London, UK; 38 Department of Ophthalmology, University of Ulm, Ulm, Germany; 39 Department of Ophthalmology and Visual Sciences, University of Alabama at Birmingham, Alabama, AL USA; 40 John A. Moran Eye Center, University of Utah, Salt Lake City, UT USA

**Keywords:** Clinical trial design, Translational research

## Abstract

This study aimed to determine the retest variability of quantitative fundus autofluorescence (QAF) in patients with and without age-related macular degeneration (AMD) and evaluate the predictive value of patient reliability indices on retest reliability. A total of 132 eyes from 68 patients were examined, including healthy individuals and those with various stages of AMD. Duplicate QAF imaging was conducted at baseline and 2 weeks later across six study sites. Intraclass correlation (ICC) analysis was used to evaluate the consistency of imaging, and mean opinion scores (MOS) of image quality were generated by two researchers. The contribution of MOS and other factors to retest variation was assessed using mixed-effect linear models. Additionally, a Random Forest Regressor was trained to evaluate the extent to which manual image grading of image quality could be replaced by automated assessment (inferred MOS). The results showed that ICC values were high for all QAF images, with slightly lower values in AMD-affected eyes. The average inter-day ICC was found to be 0.77 for QAF segments within the QAF8 ring and 0.74 for peripheral segments. Image quality was predicted with a mean absolute error of 0.27 on a 5-point scale, and of all evaluated reliability indices, MOS/inferred MOS proved most important. The findings suggest that QAF allows for reliable testing of autofluorescence levels at the posterior pole in patients with AMD in a multicenter, multioperator setting. Patient reliability indices could serve as eligibility criteria for clinical trials, helping identify patients with adequate retest reliability.

## Introduction

Age-related macular degeneration (AMD) is the leading cause of severe visual impairment in high-income countries^1^. To this day, there is only limited understanding of the pathogenesis of AMD and therapies for early and intermediate stages of AMD are missing^2^ though both late stages (neovascular and atrophic AMD) have treatment options now.

The retinal pigment epithelium (RPE) plays a key role in the pathogenesis of AMD and various other retinal diseases. RPE health and disease can be clinically assessed by fundus autofluorescence imaging (FAF)^3,4^ since RPE cells accumulate intracellular granules with intrinsic fluorophores. While frequency and distribution of these granules undergo age and disease related changes, specifically in AMD, these subcellular changes can be clinically visualized via FAF. Through technological advancement, it is now possible to quantify and compare FAF levels between patients, study sites and patient visits^5^. This is achieved in quantitative autofluorescence imaging (QAF) through incorporating a scaling bar^6^ in the imaging device.

The MACUSTAR study is a European Union funded project that aims to develop and validate clinical endpoints for studies in intermediate AMD (iAMD) that can be used to demonstrate effectiveness of therapeutic approaches^7,8^. The MACUSTAR study focuses on the iAMD stage. Age-related and AMD-related changes at the posterior pole are is divided into different stages based on pathologic changes at the posterior pole and classified using clinical fundus imaging [stages: no, early, intermediate, and late (geographic atrophy/neovascular) AMD]. The iAMD stage is of particular importance as patients often remain many years in this disease stage with only mild visual impairment. Therefore, it would be highly desirable to develop novel therapeutics that intervene during this time. As such, QAF was included to the study protocol as it could potentially assess the effect of new therapies targeting the RPE. So far, studies using QAF have found reduced autofluorescence in AMD patients, and questioned the strategy of some therapeutic approaches including visual cycle modulators^9–11^. These findings suggest that maintaining AF levels could be indicative of maintenance of RPE health and even halting AMD progression. To reliably extract such information from QAF studies, the reliability of QAF measurements needs to be further defined.

To this day, there is only limited information on the retest reliability of serial QAF images^12–14^. First, the retest reliability of QAF has only been determined for the middle Delori ring (QAF 8) and information of QAF for the whole macular region remains to be investigated^15^. Second, retest reliability of QAF to date is limited on small AMD patient cohorts and not all disease stages of AMD have been investigated^13^. Third, although a major advantage of QAF is the comparison between study sites and devices, to our knowledge, this has not been investigated in AMD. Lastly, the predictive value of “patient-reliability indices” with regard to the retest reliability in the setting of QAF is unknown. This includes the predictive value of global factors affecting all regions of the macula (e.g., disease stage, visual acuity) and local factors affecting the retest reliability of the central and peripheral macula (e.g., blur and reduced signal with increasing eccentricity due to insufficient zoom). For QAF to be applicable in clinical trials, it is mandatory to be able to identify patients with a good retest reliability.

Herein, we determined the retest reliability of QAF in individuals with and without AMD from the MACUSTAR cohort. These were assessed for all disease stages of AMD and over the whole macular area as a prerequisite for the clinical significance of QAF changes over time in interventional studies. Additionally, we investigated the predictive value of patient-reliability indices for forecasting retest reliability of patients in order to identify suitable candidates for clinical trials using QAF.

## Results

### Cohort

Eighty-one eyes from 46 patients with AMD (2 early AMD, 28 iAMD, 16 late AMD) and 39 eyes of 22 healthy controls from the MACUSTAR cohort were included in the analysis (Table 1). Number of images per site was (mean ± SD) 55.6 ± 68.3. Mean BCVA was logMAR 0.16 ± 0.36 for patients [− 0.04 ± 0.02 early AMD, 0.025 ± 0.10 iAMD, 0.80 ± 0.23 late AMD (both geographic atrophy and neovascular AMD)] and logMAR − 0.06 ± 0.1 for the subjects void of AMD relevant maculopathy.

**Table 1:** Study cohort characteristics.

			Age-related macular degeneration		
	Overall	Healthy	Early	Intermediate	Late
Number of patients	68	22	2	28	16 (14 GA)
Age [years]	71.4 ± 6.9	69.1 ± 5.8	67.5 ± 2.1	70.9 ± 7.6	75.8 ± 5.6
Sex [female]	40 (59%)	14 (64%)	2 (100%)	18 (64%)	6 (38%)
BCVA^a^	0.16 ± 0.36	− 0.06 ± 0.10	− 0.04 ± 0.02	0.03 ± 0.01	0.80 ± 0.23
Lens status, % “phakic”	69%	83%	50%	66%	58%
MOS	4.48 ± 0.39	4.51 ± 0.36	4.65 ± 0.34	4.49 ± 0.40	4.39 ± 0.39

*GA* geographic atrophy, *BCVA* best-corrected visual acuity, *MOS* mean opinion score.

^a^Visual acuity is converted in logMAR. Values are reported as mean ± SD or in percent where applicable.

### Retest reliability of QAF

**Figure 1:**
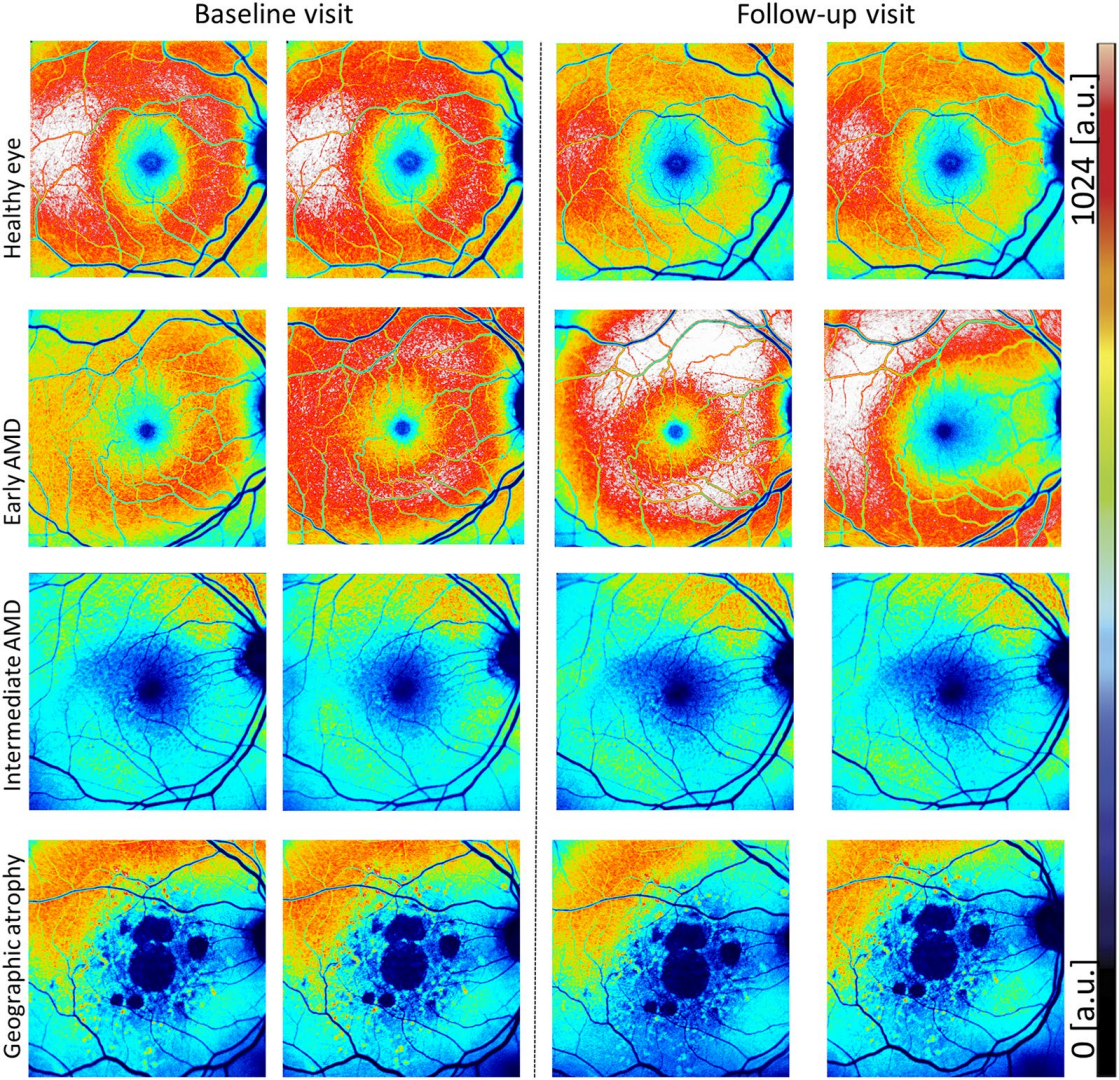
Color-coded QAF images from different AMD disease stages. Quantitative autofluorescence images (QAF) at baseline and 2-week follow-up from four study participants (male, 67 years, healthy eye; female, 69 years with early stage Age-Related Macular Degeneration (AMD); female, 75 years, intermediate AMD: male, 77 years late AMD, geographic atrophy). The color-coded images represent QAF levels. A color scale bar displaying AF level distribution is shown on the right (low QAF levels = black/blue, high QAF values = red-white). It appears that healthy and early AMD eyes have higher baseline QAF values than late disease stages of AMD. On visual inspection, same day QAF images (both columns left or right of the dashed line) appear to have a better color-coded reliability than between visits (columns compared across the dashed lines).

ICC of QAF8 (mean [95% confidence interval]) for all QAF images was 0.95 [0.93–0.96] for the intra-day and 0.79 [0.72–0.85] (Table 2), CoR as an alternate measure is reported in Table 3 for the inter-day analysis for all eyes (Fig. 1). For patients with late AMD, the ICC was slightly worse at 0.94 [0.90–0.97] for the intra-day and 0.64 [0.42–0.82] for the inter-day analysis. Excluding late AMD eyes yielded ICCs of 0.93 [0.91–0.95] (intra-day) and 0.84 [0.74–0.92] (inter-day). ICCs for all individual disease stages are reported in Table 2.

**Table 2:** Intraclass correlation coefficient of QAF8 measurements.

Disease stage	Intraclass correlation coefficient (ICC [95% CI])	
	Intra-day	Inter-day
All eyes	0.95 [0.93–0.96]	0.79 [0.72–0.85]
Healthy	0.91 [0.86–0.95]	0.70 [0.54–0.83]
Early AMD	0.96 [0.48–1.00]	N/A
Intermediate AMD	0.95 [0.92–0.97]	0.84 [0.73–0.91]
Late AMD (geographic atrophy and neovascular pooled)	0.94 [0.90–0.97]	0.64 [0.42–0.82]

Listed are the intraclass correlation coefficient (ICC) of QAF8 measurements for two clinically relevant scenarios: “Intra-day” were duplicate images acquired on the same day: “Inter-day” were images acquired approximately 2 weeks apart. Row: 1 shows ICC for all eyes, 2 for healthy only, 3 for early-only, 4 for intermediate-only and 5 for late-AMD (both GA and neovascular pooled) only.

**Table 3:** Coefficient of repeatability of QAF8 measurements.

Disease stage	Coefficient of Repeatability [a.u.]		
	Intra-day	Inter day	Inter-eye
All eyes	55.31	100.58	113.34
Healthy	68.34	127.81	126.27
Early	81.06	111.15	173.11
Intermediate	45.63	77.96	110.92
Late (geographic atrophy and neovascular pooled)	45.38	92.04	90.14

Listed are the coefficient of repeatability (CoR) of QAF8 measurements for two clinically relevant scenarios: “Intra-day” were duplicate images acquired on the same day: “Inter-day” were images acquired approximately 2 weeks apart. Row: 1 shows CoR for all eyes, 2 for healthy only, 3 for early-only, 4 for intermediate-only and 5 for late-AMD (both geographic atrophy and neovascular pooled) only.

The average inter-day ICC across all 96 segments was 0.77 [0.70–0.84]. For segments within the QAF8 ring the ICC was higher with 0.77 [0.69–0.84] and lower in peripheral segments of the QAF 97 Grid 0.74 [0.65–0.81]. Including only one eye per patient into analysis did not change ICCs noticably (Table 4).

**Table 4:** Intraclass correlation coefficient of QAF8 measurements only including only one eye per participant.

Disease stage	Intraclass correlation coefficient (ICC [95% CI])	
	Intra-day	Inter-day
All eyes	0.94 [0.92–0.96]	0.84 [0.77–0.91]
Healthy	0.92 [0.85–0.96]	0.78 [0.62–0.91]
Early AMD	N/A	N/A
Intermediate AMD	0.94 [0.9–0.97]	0.91 [0.82–0.97]
Late AMD (geographic atrophy and neovascular pooled)	0.94 [0.87–0.97]	0.71 [0.48–0.89]

Listed are the intraclass correlation coefficient (ICC) of QAF8 measurements for two clinically relevant scenarios: “Intra-day” were duplicate images acquired on the same day: “Inter-day” were images acquired approximately 2 weeks apart. Row: 1 shows ICC for all eyes, 2 for healthy only, 3 for early-only, 4 for intermediate-only and 5 for late-AMD (both geographic atrophy and neovascular pooled) only. In comparison to Table 2, only one eye per patient is included.

### Patient reliability indices

**Figure 2:**
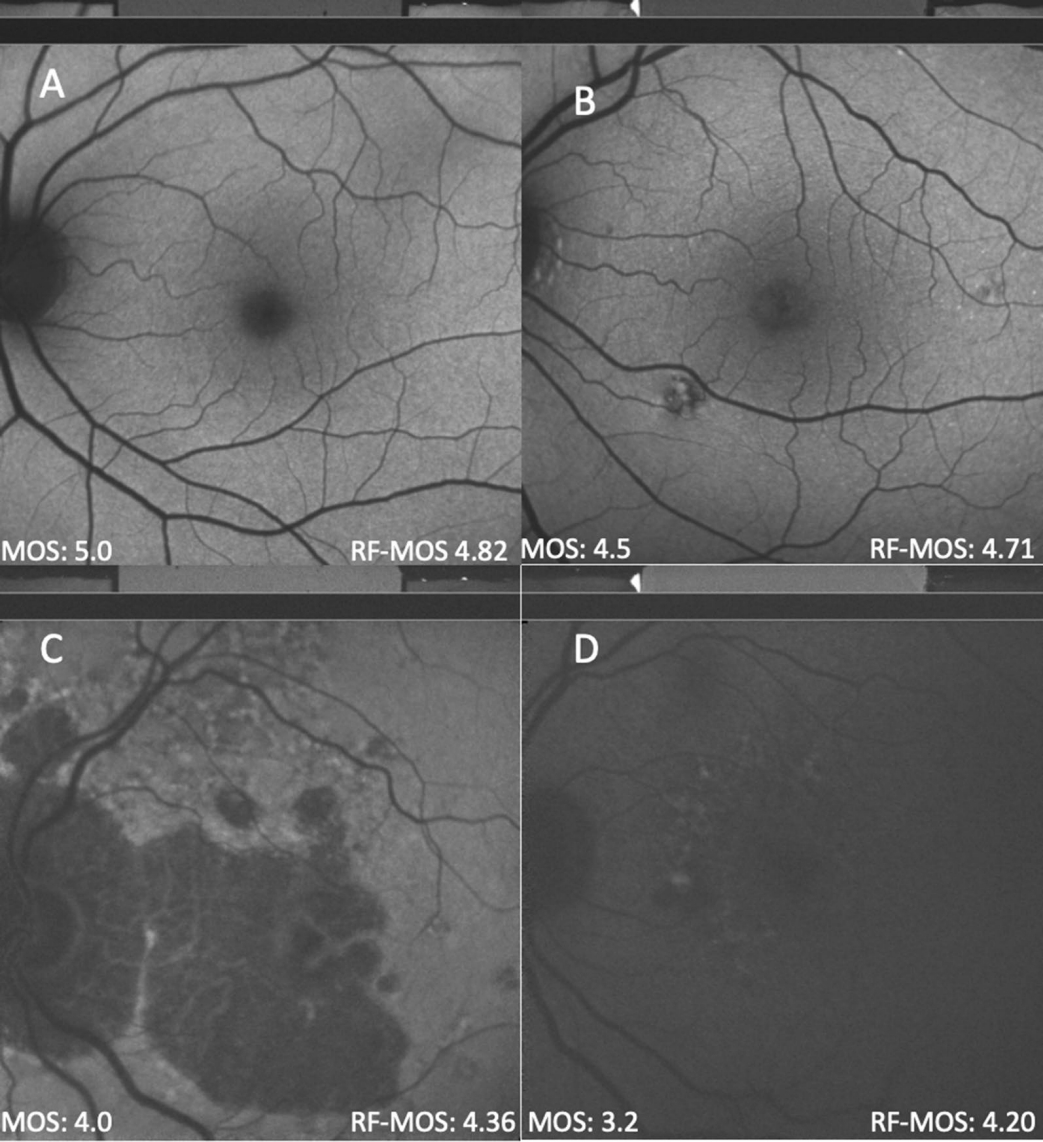
QAF image mean opinion score and predicted mean opinion score. (**A**) through (**D**) show quantitative autofluorescence (QAF) images of different quality. In the lower left corner, the Mean opinion scores (MOS) is displayed (human graders) and in the lower right the inferred Random-Forest Mean opinion score (RF-MOS) of QAF is reported. In QAF images with lower quality, the difference between MOS and RF-MOS increase. Opinion scores of QAF image quality took the following criteria into account: focus, illumination, symmetry, zoom, centering; all compiled by two readers.

**Figure 3:**
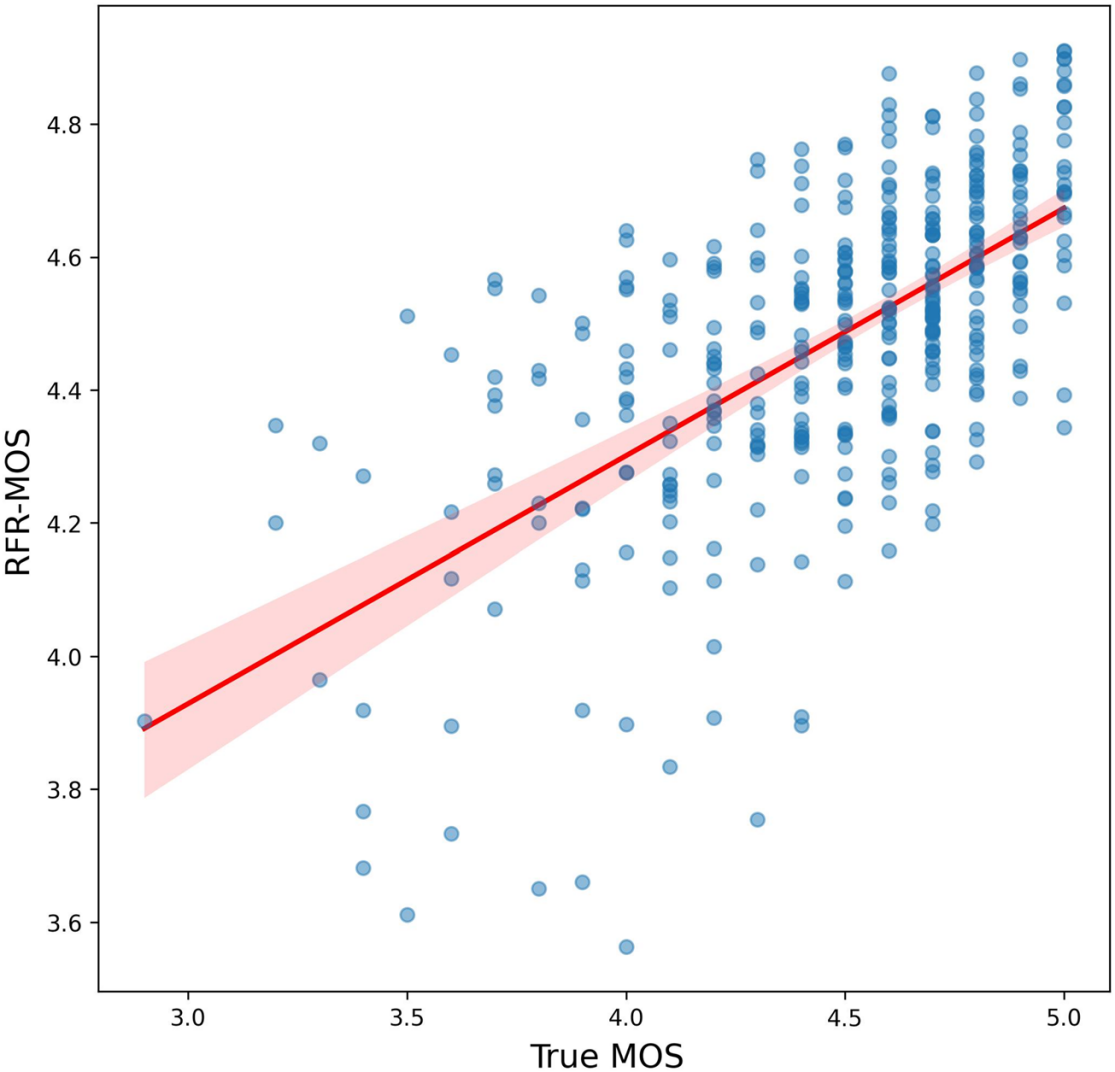
Comparison of actual vs. random forest predicted image quality scores. The scatterplot visualizes the relationship between the actual mean opinion score (MOS) of image quality on the x-axis and the predicted MOS using the random forest algorithm on the y-axis. Each point on the scatterplot represents an image. If multiple data overlap, this results in a less transparent (or darker) blue, indicating a higher density of data at that location. A red line traverses the scatterplot, representing the linear regression model's fit to the data. The light red shaded region denotes the 95% confidence interval for the regression line.

Image quality was a major driver of retest variability. Therefore, we designed a MOS of image quality, used machine learning techniques to automate image quality grading (RFR-MOS), and evaluated the effect of image quality on retest reliability in linear mixed models (Fig. 2). MOS for QAF images was 4.48 ± 0.39 overall. MOS was significantly higher in healthy (MOS of 4.51 ± 0.36) than AMD affected eyes (MOS of 4.48 ± 0.38; Mann–Whitney U p = 0.004). The RFR-MOS performed with a mean absolute error (MAE) of 0.27 **(**Fig. 3**)**. The effect of patient specific factors (age, disease status, lens status, MOS/RFR-MOS in two separate models) were evaluated with linear mixed models and are reported in Table 5. In both models, using MOS or RFR-MOS, image quality proved to be the most predictive factor for retest reliability.

**Table 5:** Results of linear mixed models.

Intra-day				
Patient reliability indices	Coefficient	Standard error	t-value	p-value
Age	0.01	0.01	0.77	0.4442
Lens status: “phakic”	− 0.21	0.18	− 1.18	0.2429
AMD disease stage				
Early	0.22	0.55	0.40	0.6933
Intermediate	− 0.54	0.18	− 2.98	**0.0044**
Late	− 0.54	0.23	− 2.38	**0.0213**
MOS	− 0.24	0.20	− 1.24	0.2166

Patient reliability indices	Coefficient	Standard error	t-value	p-value
Age	0.01	0.01	0.44	0.6587
Lens status: “phakic”	− 0.19	0.18	− 1.06	0.2943
AMD disease stage				
Early	0.14	0.54	0.26	0.7975
Intermediate	− 0.52	0.18	− 2.82	**0.0069**
Late	− 0.51	0.23	− 2.22	**0.0311**
Inferred-MOS	− 0.44	0.21	− 2.12	**0.0357**

Inter-day				
Patient reliability indices	Coefficient	Standard error	t-value	p-value
Age	− 0.00	0.02	− 0.14	0.8901
Lens status: “phakic”	− 0.21	0.22	− 0.97	0.3363
Disease stage				
Early	0.06	0.56	0.12	0.9092
Intermediate	− 0.70	0.25	− 2.78	**0.0102**
Late	− 0.29	0.28	− 1.05	0.3002
MOS	− 0.52	0.22	− 2.30	**0.0237**

Patient reliability indices	Coefficient	Standard error	t-value	p-value
Age	− 0.01	0.02	− 0.42	0.6777
Lens status: “phakic”	− 0.17	0.23	− 0.76	0.4511
Disease stage				
Early	0.03	0.58	0.05	0.9582
Intermediate	− 0.66	0.25	− 2.65	**0.0136**
Late	− 0.20	0.29	− 0.70	0.4876
Inferred-MOS	− 0.51	0.25	− 2.01	**0.0469**

Inter-eye				
Patient reliability indices	Coefficient	Standard error	t-value	p-value
Age	− 0.02	0.02	− 0.99	0.3253
Lens status: “phakic”	− 0.07	0.24	− 0.30	0.7657
Disease stage				
Early	1.39	0.80	1.73	0.0861
Intermediate	− 0.17	0.28	− 0.61	0.5472
Late	− 0.48	0.31	− 1.55	0.1274
MOS	− 0.47	0.26	− 1.82	0.0724

Patient reliability indices	Coefficient	Standard error	t-value	p-value
Age	0.02	0.02	− 1.21	0.2301
Lens status: “phakic”	− 0.05	0.25	− .021	0.8335
Disease stage				
Early	1.11	0.80	1.39	0.1679
Intermediate	− 0.14	0.28	− 0.48	0.6311
Late	0.40	0.31	− 1.28	0.2057
Inferred-MOS	− 0.54	0.28	− 1.96	0.0530

Result of the six linear mixed effect models performed in this study (two for each scenario: intra-day [duplicate image same day], inter-day [images acquired 2 weeks apart] and inter-eye [comparison of left and right eye] for both the mean opinion score graded by human readers and inferred from machine learning are summarized. Each row shows the coefficient, standard error, t-value and p-value of each fixed effect. Statistically significant p-values (p< 0.05) are marked bold.

### Retest reliability of identified “eligible images”

As a model for clinical trial criteria, we chose a combination of patient reliability indices that (i) are easily and objectively determinable and (ii) offer valuable information about retest reliability. As such, we chose the following criteria: MOS of ≥ 4.5 and only included healthy, early- and iAMD participants (see paragraph patient reliability indices and Table 5). We further included only the QAF8 values as they proved to be most reliable in preceding analyses^14^. After applying the quality criteria, inter-day ICC improved from 0.79 to 0.84 [0.74–0.92]. We further provided the ICC for intra- and inter-day variability of QAF retest-reliability for alternate clinical trial criteria (Table 6) to ensure a good balance between data availability and retest-reliability requirements. For example, reducing the MOS to ≤ 3.5 with all other criteria constant, deteriorated the inter-day ICC to 0.8 [0.7–0.88].

**Table 6:** Intraclass correlation coefficients (ICC).

Scenario	Criteria used/patient reliability indices			Results: ICC	
	Cohort	MOS	Grid	Intra-day	Inter-day
1	AMD only, excluding late AMD	≥ 4.0	QAF8	0.96 [0.94–0.97]	0.85 [0.77–0.91]
2	AMD only, excluding late AMD	≥ 3.5	QAF8	0.95 [0.93–0.97]	0.8 [0.70–0.88]
3	AMD only, including late AMD	≥ 4.5	QAF8	0.96 [0.93–0.97]	0.88 [0.77–0.95]
4	AMD only, excluding late AMD	≥ 4.5	QAF 97	0.96 [0.93–0.97]	0.87 [0.76–0.95]

This table lists the intraclass correlation coefficients (ICC) for same day and 2 weeks follow-up evaluation for different samples of possible inclusion criteria that could be applied in clinical studies.

*AMD* age-related macular degeneration, *MOS* mean opinion score of image quality grading, *ICC* intraclass correlation coefficient.

## Discussion

This study provides retest-reliability of QAF imaging values for same-day and 2-week follow up visits. QAF image quality, as assessed by either human graders or random forest regression, was most predictive of retest variability. These findings provide important insights into the reliability of reported QAF values and patient selection for studies including QAF imaging as an endpoint.

### Retest reliability

Proper repeatability and reliability as well as consistent follow-up agreement are a prerequisite for investigating possible changes in QAF in longitudinal studies as they yield the best chance to detect a true effect/change. So far, reported retest reliability has varied heavily. In healthy eyes, retest has been reported as ± 6–± 11% for same day and ± 7% to ± 14% for inter-day variability^6,15,16^. In monocentric studies of retinal diseases, QAF retest reliability was reported slightly lower but nonetheless excellent: ± 10.3% in recessive Stargardt disease (same day)^14^, ± 7% in Best vitelliform macular dystrophy (same day), ± 18.1%–± 20.2% (inter-day) AMD^17,18^. First real-life multi-center results from an interventional study in Stargardt disease, however, showed a higher retest variability of ± 26.1% (same day) and ± 40.3% (inter-day)^19^, respectively. Possible reasons for this deviation are demanding imaging protocol and operator variability, among others^20^. The reported results are in line with our results of ± 10.0% (same day) and ± 18.9% (inter-day), respectively.

Our results confirm the notion that QAF is substantially more challenging in a multicenter study. We, therefore, also propose methods of patient selection and QAF measurement techniques in this study, to improve the reliability of measurements even in the absence of large sample sizes. Additionally, improved staff training may lead to improved results. Future studies should compare retest reliability in relation to imaging staff experience.

Reiter and colleagues^13^ also investigated differences in QAF values in healthy and AMD patients for the different rings of the Delori pattern, and found that the middle eight-segment ring achieved best reproducibility. Similarly, we investigated retest reliability for individual segments, and could corroborate that the segments related to the QAF8 were associated with better retest reliability than more peripheral segments. This should be taken into consideration in future studies analyzing QAF outside of the border of the Delori grid, and especially in the near-periphery. We suspect worse retest reliability in the border-zone of the QAF image due to shadowing effects of the eyelid and/or insufficient zoom during image acquisition.

### Predicted image quality

To our knowledge, this is the first study analyzing the effects of image quality on QAF measurements and retest reliability. In other imaging modalities (e.g., OCT or OCT angiography), image quality assessment is already routinely used in clinical studies^21–23^. Most metrics for image quality assessment in image processing applications rely on a sensitivity-based framework (e.g., peak signal-to-noise ratio)^24–26^. However, the downside in such an approach is that pathology is falsely classified as deteriorated image quality. For example, a peak signal-to-noise ratio will differ strongly if the RPE is missing like is the case in geographic atrophy (peak signal vanished). We, therefore, aimed on developing an objective image quality metric that correlates with perceived quality measurement. Our RF-MOS was trained on a human-based opinion score and strongly correlates with perceived image quality. Replacing manual image grading by an automated assessment would nonetheless have several advantages, apart from saving time: image quality assessment would become less prone to human error, and more reproducible (and thus comparable between studies)^27^.

Table 6 can assist investigators in selecting cut-off values for image-quality while accounting for disease status, study design and the QAF Grid utilized. Through automated image quality assessment, the expected ICC´s will match the results of this study to a higher degree than would be feasible through human grading.

### Patient reliability indices

Patient reliability indices have a long-standing history in ophthalmology and originally stem from glaucoma^25,26^. In glaucoma management, visual field assessment is extremely important but also dependent on patient’s performance. Here false-positive error, fixation loss and other indicators can determine the reliability of visual field testing in a patient^28^. In imaging, these indices are currently not being used routinely, but may be beneficial in more challenging modalities such as QAF. Our finding was that only image quality had a significant effect on retest reliability. Retest reliability between the different disease stages did not prove to be statistically different (albeit slightly lower values for late AMD were found)^24,29–32^. These results suggest that QAF is feasible in all AMD disease stages.

Given the limited number of patients outside of the iAMD group, these results have to be interpreted cautiously. Reiter and colleagues found a higher retest reliability in AMD patients (ICC 0.93 with retinal changes/ICC 0.96 without retinal changes)^13^ than in control participants. For interventional studies utilizing QAF, we propose criteria to ensure a high reliability of QAF imaging.

### Limitations and strengths

Some reliability indices such as the skill level of the operator could not be evaluated. Furthermore, the dataset was skewed with a limited number of patients in the early and late AMD categories. Finally, additional information on the lens status (e.g., cataract score, QAF of the lens, lenticular nuclear density) could have added insight into the effect of the ageing lens on retest reliability^33–35^. The order of the imaging protocol and time of day was not mandatory; therefore, patient fatique during the imaging session might also affect QAF retest reliability. Finally, the inclusion of both eyes from one participant to determine the ICC values disregards the hierarchical structure of the data. We, therefore, further report ICC values including only a random of each participant in Table 4. However, strengths of this study include the multicenter design and having both duplicate same day and 2-week follow-up images in a large cohort of both AMD-affected and healthy participants that were well characterized with multimodal imaging. Furthermore, novel elements in this study are the use of patient reliability indices to identify patient cohorts with good retest reliability as well as subjective and machine learning based image quality assessment.

## Conclusions

In conclusion, QAF retest reliability for iAMD patients was good, higher for same day than different day repeats. Image quality, assessed by human or automated grading, is the major driver of retest variability. Based on our results we propose solutions for patient selection to augment retest reliability and pave the way for QAF inclusion in future interventional clinical trials.

## Methods

In the prospective European MACUSTAR study, participants with iAMD and neighboring disease stages (early AMD, late AMD) as well as healthy controls were clinically evaluated with multimodal imaging and functional testing for a study period of 3 years^8,36^. For the current analysis, images from the cross-sectional arm of the MACUSTAR clinical study with available QAF images (6 study sites, 120 participants) were included. This study was conducted and analyzed in compliance with the Declaration of Helsinki and according to the standards of good clinical practice. This study was approved by the EMA, US FDA, and NICE, and participants signed written informed consent before study inclusion^7^. The study was further approved by the local ethic committees of the University Hospital Bonn ethics committee (384/17), Paris Ouest IV (04/18_2), AIBILI (032/2017/AIBILI/CE), Nova Medical School (13507/2017), London Queen Square Research Ethics Committee (18/LO/0145), Center for Sundhed Glostrup (H-18000126), Comitato Etico Milano (37910/2018), Ospedale San Raffaele (dated 25/10/2018), Radboudumc technology center (2017-3954) and LUMC commissie medische ethiek (L18.055/SH/sh).

Inclusion and exclusion criteria of the MACUSTAR study have been reported elsewhere^7^. Briefly, subjects aged 55–85 at baseline, AMD (with the largest cohort being iAMD) or healthy eyes and the absence of other eye disorders were included^36^. iAMD was defined as bilateral large drusen and/or pigment abnormalities or extrafoveal geographic atrophy in the partner eye (for a full list of AMD disease stage criteria see Table 1 in Terheyden et al.^36^). Additional exclusion criteria from the MACUSTAR requirements for the current study were the non-availability of QAF images at baseline and 2-week follow up visit, insufficient image quality (see assessment below) for image analyses, and a high degree of lens opacification. Certified staff at the individual study sites acquired all multimodal images (including but not limited to color/multicolor fundus photography, optical coherence tomography OCT, green FAF, blue FAF) as well as QAF images. Retinal imaging including QAF imaging was performed by certified technicians and on certified equipment. Retinal imaging was assessed after administration of mydriatic eye drops (e.g., 2.5% phenylephrine, 0.5% tropicamide). The order of image acquisition and specific time of day was not mandatory but guidelines were provided to the study sites. From the MACUSTAR assessment of functional endpoints (including but not limited to fundus controlled perimetry, low luminance acuity, Moorefield’s acuity test, dark adaptation contrast sensitivity and performance based tests) only the best corrected visual acuity was used in this study. Best-corrected visual acuity was assessed by certified personnel using standard ETDRS charts and converted to logMAR for analysis^7^.

### Image analysis

QAF images were provided by the central reading center of the MACUSTAR study (GRADE Reading Center, Bonn, Germany). As described previously, custom written FIJI plugins (“https://sites.imagej.net/CreativeComputation/”) were used for QAF analysis^12^. Briefly, using landmark correspondences (e.g., vessel bifurcations), images were registered to SD-OCT images to ensure aligned QAF measurements (equal rotation and uniform scaling). Next, for QAF analysis grid positioning, the foveola (maximal foveal depression and rise of external limiting membrane) and the closest edge of the optic nerve head were marked in corresponding OCT scans.

QAF images were then post-processed and adjusted for the device-specific reference calibration factor as provided by the manufacturer, as well as subject's age. Finally, QAF images were converted to colored 8-bit images, with QAF values limited to 0–511 [QAF a.u.]. The QAF97 grid used bisects each original QAF ring segment (and results used for the eccentricity analysis), resulting in a total of 97 segments^6^ (Supplemental Figs. 1 and 2). Further, the QAF 8 (mean of middle Delori ring) was used and reported as this was the most common outcome measure in other QAF studies^6^. For each segment, the mean, maximum and minimum QAF values, standard deviation of QAF values, and the number of pixels of the analyzed area were exported.

To further analyze the effect of QAF image quality on retest reliability, opinion scores of QAF images were gathered. Opinion scores of QAF image quality (focus, illumination, symmetry, zoom, centering) were compiled by two trained medical readers (LvdE, MM) and averaged to yield mean opinion scores (MOS). Grading was performed masked to each other. Images were graded on a semi-qualitative scale between 0 and 5 and the mean of all criteria was computed.

### Statistical analysis

Statistical analyses were performed in Python (notably using the scikit-learn^37^ and Pingouin^38^ packages) and R using the lmerTest^39^ and MuMin^40^ packages. To quantify retest variability, the Intraclass Correlation Coefficient (ICC) as defined by Shrout and Fleiss^20^, and the repeatability coefficient (RC), computed as outlined by Bland and Altman^41^ via intra-subject standard deviations, were used.

ICCs were evaluated between duplicate images at one visit (intra-day) and between images at baseline and 2-week follow up (inter-day), for all four images separately.

Visual acuity was converted to the logarithm of the Minimum Angle of Resolution (logMAR). To consider the association between MOS and retest variability, we utilized linear mixed-effect models to account for intra-subject correlation, with nested random effects for study site and patient. Age, lens status and disease stage were included as categorical fixed effects.

For MOS prediction, we used a Random Forest Regressor (RFR), as implemented by scikit-learn, with 200 estimators, no bootstrapping, and otherwise the default hyperparameters^42^. As predictors, the lens status, age at baseline, and each segment value of the QAF 96 grid was used. These validation MOS predictions were then used to repeat the mixed-effect model analysis with RFR-MOS in place of the true MOS.

### Supplementary Information

Supplementary Legends.

Supplementary Figure 1.

Supplementary Figure 2.

## Data Availability

The datasets used and/or analyzed during the current study available from the corresponding author on reasonable request.
