# Supplementary material for: Retest variability and patient reliability indices of quantitative fundus autofluorescence in age-related macular degeneration: a MACUSTAR study report
Source: Sci Rep. 2023 Oct 13;13:17417. doi: 10.1038/s41598-023-43417-y (PMC10576044; doi:10.1038/s41598-023-43417-y)
Supplement: Supplementary file 1 — Supplementary Legends. [file 41598_2023_43417_MOESM1_ESM.docx]

**Supplement Figure 1: QAF Grids.** Quantitative autofluorescence image of a right eye from a patient with intermediate AMD. The Figure shows the QAF8 (blue semitransparent area) in A, and QAF 9 grid in B that were used in this study to investigate the association of retest reliability with eccentricity.

**Supplementary figure 2:** Supplementary figure 2 visualizes included (green, bottom left) and excluded quantitative autofluorescence (QAF) images. The top right corner shows the disease status and a representative QAF image. Reasons for exclusion were if important features such as the optic disc, fovea and/or inferior/superior arcade were not depicted, or if the image was not sufficiently lit or out of focus to detect key features. As this study investigated the impact of image quality on retest-reliability inclusion criteria were more lenient than in comparative studies.
