# Supplementary figures and images for: Retest variability and patient reliability indices of quantitative fundus autofluorescence in age-related macular degeneration: a MACUSTAR study report
Source: Sci Rep. 2023 Oct 13;13:17417. doi: 10.1038/s41598-023-43417-y (PMC10576044; doi:10.1038/s41598-023-43417-y)

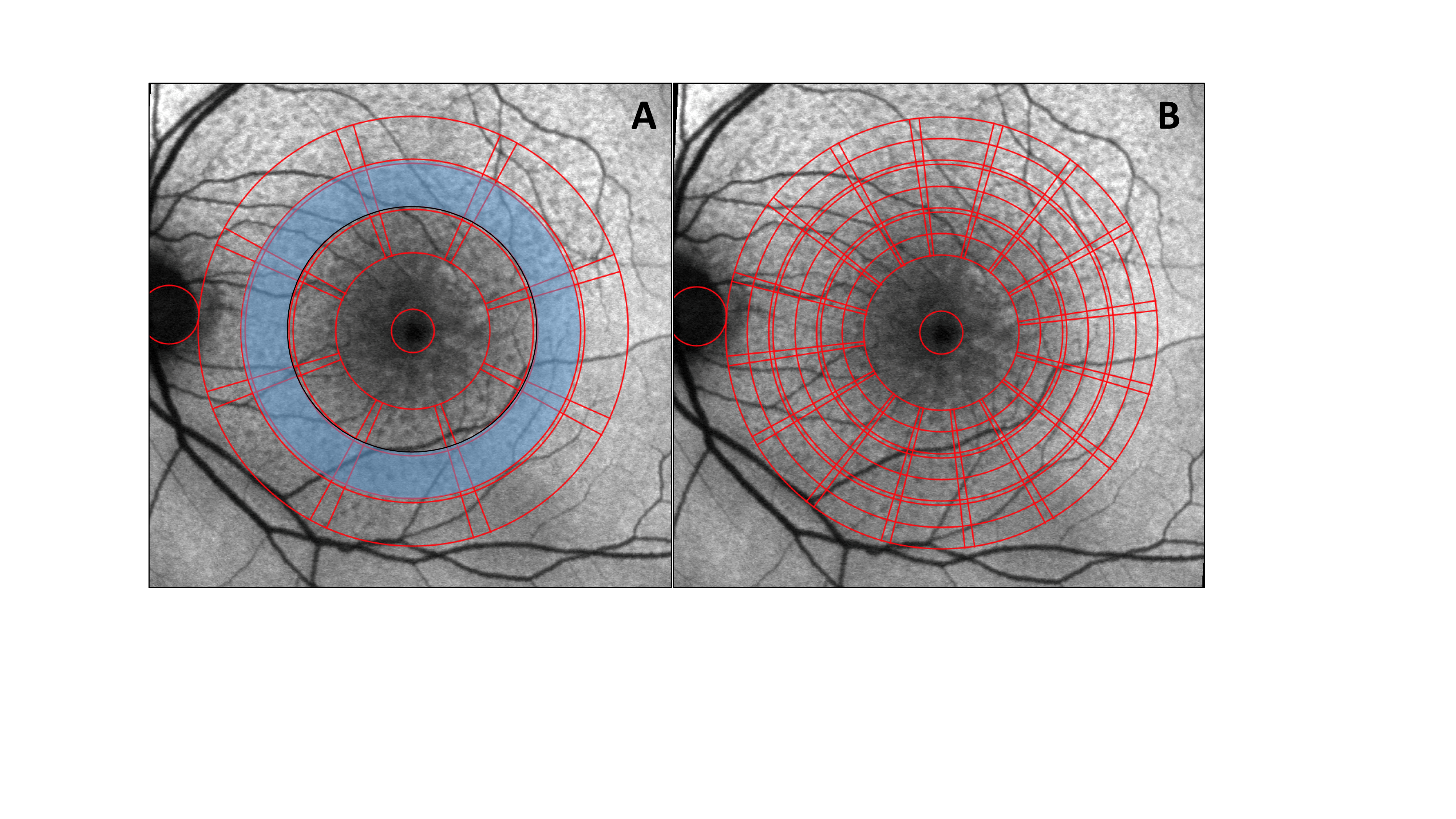

Supplement: Supplementary file 2 — Supplementary Figure 1. [file 41598_2023_43417_MOESM2_ESM.png]

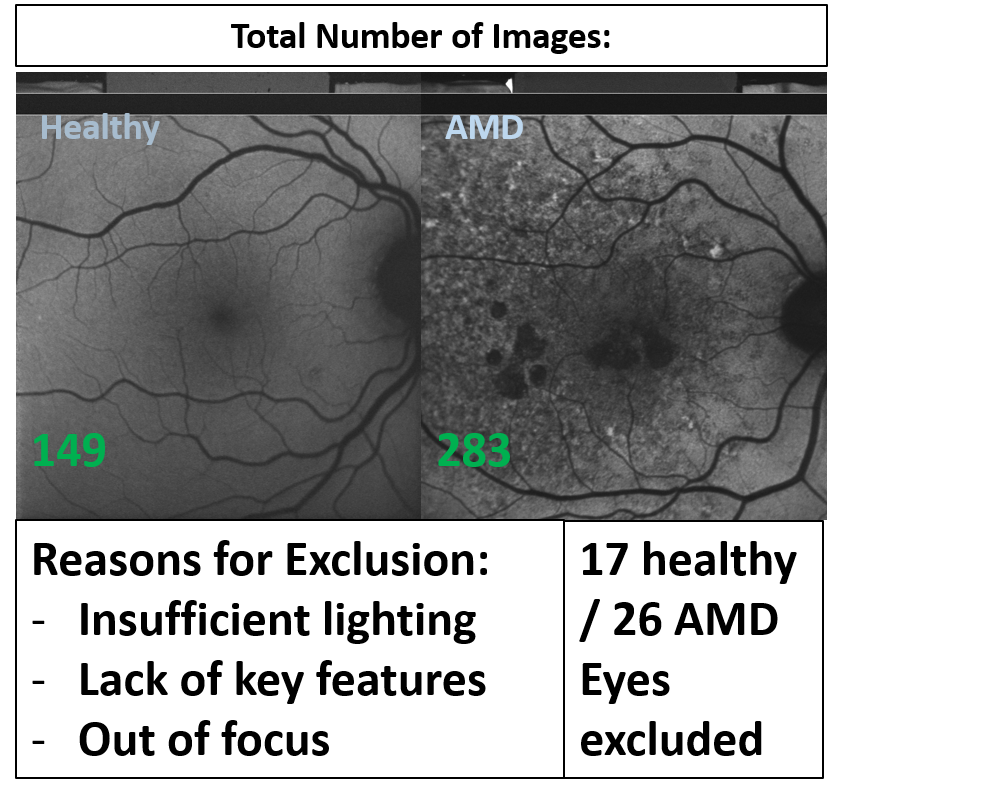

Supplement: Supplementary file 3 — Supplementary Figure 2. [file 41598_2023_43417_MOESM3_ESM.png]
